# Standard dosing of enoxaparin versus unfractionated heparin in critically ill patient with COVID-19: a multicenter propensity-score matched study

**DOI:** 10.1186/s12959-022-00432-9

**Published:** 2022-12-08

**Authors:** Khalid Al Sulaiman, Ohoud Aljuhani, Ghazwa B. Korayem, Awatif Hafiz, Mai Alalawi, Hisham A. Badreldin, Ali F. Altebainawi, Ramesh Vishwakarma, Abdulrahman Alissa, Albandari Alghamdi, Abeer A. Alenazi, Huda Al Enazi, Shahad Alanazi, Abdullah Alhammad, Jahad Alghamdi, Mashael AlFaifi, Faisal A. Al Sehli, Maram A. Aldossari, Alaa A. Alhubaishi, Anfal Y. Al-Ali, Hasan M. Al-Dorzi

**Affiliations:** 1 Pharmaceutical Care Department, King Abdulaziz Medical City (KAMC) - Ministry of National Guard Health Affairs (MNGHA), Riyadh, Saudi Arabia; 2 College of Pharmacy, King Saud Bin Abdulaziz University for Health Sciences, Riyadh, Saudi Arabia; 3 King Abdullah International Medical Research Center (KAIMRC), PO Box 22490, 11426 Riyadh, Saudi Arabia; 4 Saudi Critical Care Pharmacy Research (SCAPE) Platform, Riyadh, Saudi Arabia; 5 Department of Pharmacy Practice, Faculty of Pharmacy, King Abdulaziz University, Jeddah, Saudi Arabia; 6 Department of Pharmacy Practice, College of Pharmacy, Princess Nourah bint Abdulrahman University, P.O. Box 84428, Riyadh, 11671 Saudi Arabia; 7 Department of Pharmaceutical Sciences, Fakeeh College for Medical Sciences, Jeddah, Saudi Arabia; 8 Pharmaceutical Care Services, King Khalid Hospital, Hail Health Cluster, Hail, Saudi Arabia; 9 Norwich Medical School, University of East Anglia, Norwich, United Kingdom; 10 Pharmaceutical Care Services, King Abdulla bin Abdulaziz University Hospital, Riyadh, Saudi Arabia; 11 Pharmaceutical Care Department, Prince Sultan Military Medical City, Riyadh, Saudi Arabia; 12 Department of Clinical Pharmacy, College of Pharmacy, King Saud University, Riyadh, Saudi Arabia; 13 Saudi Food and Drug Authority, Riyadh, Saudi Arabia; 14 Pharmaceutical Care Department, Dhahran Eye Specialist Hospital, Dhahran, Saudi Arabia; 15 Intensive Care Department, King Abdulaziz Medical City, Riyadh, Saudi Arabia

**Keywords:** COVID-19, SARS-Cov-2, DVT prophylaxis, Enoxaparin, Unfractionated Heparin, Critically ill, Intensive Care Units (ICUs), Thrombosis, Bleeding, Mortality

## Abstract

**Background:**

Thrombotic events are common in critically ill patients with COVID-19 and have been linked with COVID-19- induced hyperinflammatory state. In addition to anticoagulant effects, heparin and its derivatives have various anti-inflammatory and immunomodulatory properties that may affect patient outcomes. This study compared the effectiveness and safety of prophylactic standard-doses of enoxaparin and unfractionated heparin (UFH) in critically ill patients with COVID-19.

**Methods:**

A multicenter, retrospective cohort study included critically ill adult patients with COVID-19 admitted to the ICU between March 2020 and July 2021. Patients were categorized into two groups based on the type of pharmacological VTE thromboprophylaxis given in fixed doses (Enoxaparin 40 mg SQ every 24 hours versus UFH 5000 Units SQ every 8 hours) throughout their ICU stay. The primary endpoint was all cases of thrombosis. Other endpoints were considered secondary. Propensity score (PS) matching was used to match patients (1:1 ratio) between the two groups based on the predefined criteria. Multivariable logistic, Cox proportional hazards, and negative binomial regression analysis were used as appropriate.

**Results:**

A total of 306 patients were eligible based on the eligibility criteria; 130 patients were included after PS matching (1:1 ratio). Patients who received UFH compared to enoxaparin had higher all thrombosis events at crude analysis (18.3% vs. 4.6%; *p*-value = 0.02 as well in logistic regression analysis (OR: 4.10 (1.05, 15.93); *p*-value = 0.04). Although there were no significant differences in all bleeding cases and major bleeding between the two groups (OR: 0.40 (0.07, 2.29); *p*-value = 0.31 and OR: 1.10 (0.14, 8.56); *p*-value = 0.93, respectively); however, blood transfusion requirement was higher in the UFH group but did not reach statistical significance (OR: 2.98 (0.85, 10.39); *p*-value = 0.09). The 30-day and in-hospital mortality were similar between the two groups at Cox hazards regression analysis. In contrast, hospital LOS was longer in the UFH group; however, it did not reach the statistically significant difference (beta coefficient: 0.22; 95% CI: -0.03, 0.48; *p*-value = 0.09).

**Conclusion:**

Prophylactic enoxaparin use in critically ill patients with COVID-19 may significantly reduce all thrombosis cases with similar bleeding risk compared to UFH.

## Introduction

Since the outbreak of the novel coronavirus disease 2019 (COVID-19) worldwide, the attention initially was focused on its pulmonary complications [1–3]. Nonetheless, patients with COVID-19 also have non-pulmonary complications that could lead to increased morbidity and mortality. These complications include thrombotic complications and end organ damage [3]. COVID-19-associated thrombotic complications are assumed to be similar to the systemic coagulopathy that occurs during severe infections, commonly known as sepsis-induced coagulopathy (SIC) or disseminated intravascular coagulation (DIC). Coagulopathy in patients with severe COVID-19 are usually characterized by D-dimers and fibrinogen levels elevation, mild prolongation of prothrombin time (PT), and thrombocytopenia.Whereas patients with DIC have decreased fibrinogen levels, severe thrombocytopenia, and significant PT prolongation [4, 5]. These abnormalities in COVID-19 are based on the inflammatory state and the speed at which treatment commences particularly if the patient has been exposed to drugs such as corticosteroids, antiviral therapy, and monoclonal antibodies These changes may explain the differences in clinical severity and mortality in patients with COVID-19 [6].

The incidence of venous thromboembolism (VTE) in patients with severe COVID-19 infection reaches 25% with even higher rates in critically ill patients ranging between 20 and 49% [7, 8]. All critically ill patients are at high risk of thrombosis, thus thromboprophylaxis is indicated for them. The optimal dose of anticoagulants for critically ill patients with COVID-19 was debated earlier in the pandemic. However, several randomized clinical trials (RCTs) showed no additional benefit for using higher than the standard thromboprophylaxis dose in critically ill patients with COVID-19 [9]. Therefore, the current guidelines recommend using standard prophylaxis dose of low molecular weight heparin (LMWH) or unfractionated heparin (UFH) for VTE prophylaxis in critically ill patients with COVID-19 [10–12].

LMWH and UFH are commonly used for thromboprophylaxis. Both have been shown to be as efficacious in preventing VTE in hospitalized patients [13, 14]. However, some studies suggested that LMWH was more effective than UFH [15, 16]. In critically ill patients, the evidence suggests that LMWH for thromboprophylaxis might be superior to UFH [17]. However, there is no clear evidence about the benefit of UFH over LMWH or vice versa in critically ill patients with COVID-19. A retrospective observational study included moderately ill and severely ill patients with COVID-19 found that enoxaparin was associated with lower 28-day mortality compared to UFH when used for VTE treatment or prevention [18]. On the other hand, a RCT including critically ill patients with COVID-19 reported significantly increased risk of intubation and mortality in patients receiving prophylactic dose of enoxaparin compared to therapeutic dose of UFH [19]. The decision about the most effective thromboprophylaxis regimen in critically ill patients with COVID-19 remains questionable. In addition to anticoagulant effects, heparin and its derivatives have various anti-inflammatory and immunomodulatory properties, but enoxaparin has more impact on the inflammatory markers than UFH [20]. Thus, the aim of this study was  to compare the effectiveness and safety of prophylactic standard-doses of enoxaparin and UFH in critically ill patients with COVID-19.

## Methods

### Study design and participants

**Fig. 1:**
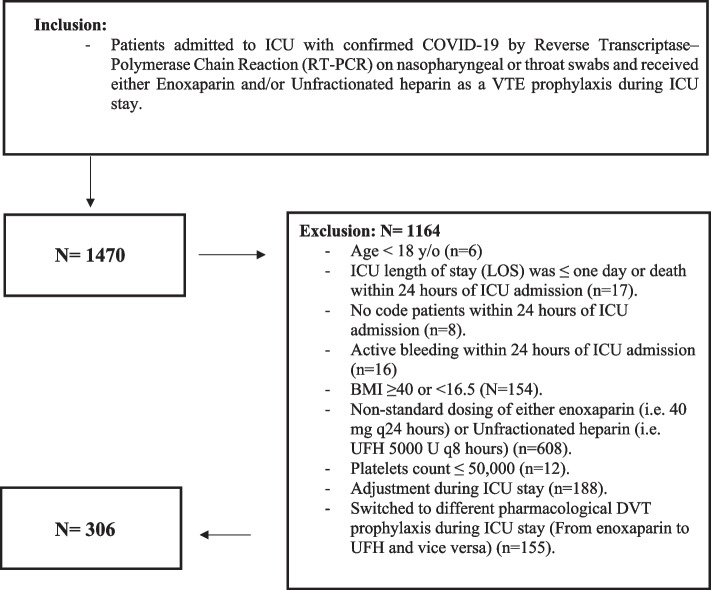
Eligibility criteria flowchart

This was  a multicenter, retrospective cohort study that included critically ill adult (age ≥ 18 years) patients with confirmed COVID-19, who were admitted to the intensive care units (ICUs) from March 01, 2020, until July 31, 2021, and received pharmacological VTE prophylaxis at standard dosing (Unfractionated heparin (UFH) 5000 units subcutaneously every 8 h versus enoxaparin 40 mg subcutaneously every 24 h) throughout their ICU stay. Reverse Transcriptase-Polymerase Chain Reaction (RT-PCR) on nasopharyngeal or throat swabs was used to diagnose COVID-19. Patients were excluded if they had active bleeding within 24 h of ICU admission, platelets count ≤ 50,000 10^9/l, BMI ≥ 40 or < 16.5 kg/m2, were not on standard dosing of either enoxaparin or UFH, switched to a different type of pharmacological DVT prophylaxis during ICU stay (from enoxaparin to UFH and vice versa), underwent dose adjustment for pharmacological VTE prophylaxis during ICU stay, ICU length of stay (LOS) ≤ one day, died within the first 24 h of ICU admission or were labeled as “Do-Not-Resuscitate” (Fig. 1). All patients were followed until they were discharged from the hospital or died during their in-hospital stay. The study was approved by the Institutional Review Board (IRB)—King Abdullah International Medical Research Center (KAIMRC) (Ref.# NRC22R/088/02). Informed consent from the study patients was waived due to the retrospective observational nature of the study. All methods were performed in accordance with relevant guidelines and regulations.

### Study setting

This study was conducted at five hospitals in Saudi Arabia: King Abdulaziz Medical City (Riyadh & Jeddah), King Abdulaziz University Hospital (Jeddah), King Abdullah bin Abdulaziz University Hospital (KAAUH) (Riyadh), and King Salman Specialist Hospital (Hail). The selection of these centers was based on the geographic distribution, availability of electronic health records, and the center’s willingness  to participate in the national project. The primary site for this multicenter study was King Abdulaziz Medical City (Riyadh).

### Data collection

Data were collected using Research Electronic Data Capture (REDCap®) software hosted by King Abdullah International Medical Research Center (KAIMRC). Demographic data included: comorbidities, vital signs and laboratory tests, severity score (Acute Physiology and Chronic Health Evaluation II (APACHE II) [21], acute kidney injury (AKI), use of prone positioning, and mechanical ventilation (MV) parameters (e.g., lowest PaO_2_/FiO_2_ ratio, highest FiO_2_ requirement) within 24 h of ICU admission. Furthermore, within 24 h of ICU admission, a renal profile, liver function tests, coagulation profile (i.e., International normalized ratio (INR), activated partial thromboplastin time (aPTT), fibrinogen, D-dimer), and other markers (ferritin, procalcitonin, and creatine phosphokinase) were collected. The type, initial dose, and adjustment dose of pharmacological VTE prophylaxis were recorded. The use of tocilizumab, and corticosteroids were also documented.

### Outcomes

The primary endpoint was all cases of thrombosis. The secondary endpoints were all cases of bleeding, major bleeding, 30-day and in-hospital mortality, hospital LOS, ICU, ventilator-free days (VFDs) at 30 days, and thrombocytopenia (i.e., heparin induced thrombocytopenia (HIT) and heparin associated thrombocytopenia (HAT)). Moreover, follow-up biomarkers were considered secondary outcomes during ICU stay, such as C-reactive protein (CRP), D-dimer, Fibrinogen, and ESR levels.

### Definitions of outcomes

• All cases of thrombosis (arterial and venous) were defined using the International Statistical Classification of Diseases (ICD)10-CM code, chart documentation (i.e., Myocardial infarction (MI), ischemic stroke, pulmonary embolism, deep vein thrombosis) and/or radiological studies including ultrasound and computed tomography scans [22].
• The 30-day mortality was defined as a death occurring for any cause within 30 days of the admission date during hospital stay; patients who were discharged from the hospital alive before 30 days were presumed to be survivors.
• VFDs at 30 days were calculated as the following: if the patients die within 30 days of MV, the VFDs = 0, VFDs = 30 − days after MV initiation (if patient survived and was successfully liberated from MV), and VFDs = 0 if the patient is on MV for >30 days.
• AKI was defined as a sudden decrease of renal function within 48 hours, defined by an increase in absolute serum creatinine of at least 26.5 μmol/L (0.3 mg/dL) or by a percentage increase in serum creatinine ≥ 50% (1.5× baseline value) during ICU stay [23].
• Heparin-induced thrombocytopenia (HIT) is an adverse drug reaction mediated by platelet-activating antibodies that target complexes of platelet factor 4 and heparin. HIT was confirmed by using platelet factor 4 (PF4) antibody level in the blood. [24]
• Heparin-associated thrombocytopenia (HAT) is nonimmune HAT that is characterized by a mild, transient decline in platelet count that occurs one to four days after initiating heparin; it is relatively benign and usually resolves spontaneously despite the continuation of heparin. [25]
• Major bleeding was defined according to the International Society on Thrombosis and Hemostasis (ISTH) definition. Any bleeding not fulfilling the criteria of major or clinically significant bleeding was identified as a minor bleed. [26]

### Statistical analysis

We reported continuous variables as mean and standard deviation (SD), or median with lower (Q1) and upper (Q3) quartile. In contrast, categorical variables were reported as numbers with percentages as applicable. The normality assumptions for all continuous variables were evaluated using a statistical test (the Shapiro–Wilk test) and graphical representation (i.e., histograms and Q-Q plots). We utilized the Chi-square or Fisher's exact test for categorical variables. We used the student t-test to compare normally distributed continuous variables and the Mann–Whitney U test for non-normally distributed continuous variables. The baseline characteristics of the two study groups were compared.

Propensity score (PS) matching procedure (Proc PS match) (SAS, Cary, NC) was used to match patients (1:1 ratio) who received UFH (active group) to patients who received enoxaparin prophylaxis dose (control group). These PS scores were generated through propensity score analysis based on clinically and statistically relevant covariates: age, APACHE II score, D-dimer level, chronic kidney disease as comorbidity, AKI status within 24 h of ICU admission for all the outcomes considered in this study. The patients were matched only if the difference in the logits of the propensity scores for pairs of patients from the two groups was less than or equal to 0.7 times the pooled estimate of the standard deviation. A greedy nearest neighbor matching method was used in which one patient who received UFH (active) matched with one patient in the enoxaparin group (control), which eventually produced the smallest within-pair difference among all available pairs with treated patients.

Regression analysis was done for the study outcomes after considering PS scores as covariates in the model. The hazard ratios (HR), odds ratios (OR), or beta estimates with 95% confidence intervals (CIs) were reported as appropriate. Visual assessment was performed to assess the assumption by plotting the log(-log) plot and testing the correlation of scaled Schoenfeld residuals with rank-ordered time. The Hosmer–Lemeshow goodness-of-fit test was used to evaluate model fit. Multivariable Cox proportional hazards regression analyses were performed for the 30-day and in-hospital mortality. The proportionality assumption was assessed before fitting the Cox model. No imputation was made for missing data, as the cohort of patients in this study was not derived from random selection. We considered a* P* value of < 0.05 statistically significant and used SAS version 9.4 for all statistical analyses.

## Results

A total of 1470 patients who were admitted to ICUs with COVID-19  were screened during the study period; 306 patients were  enrolled for the present study based on the eligibility criteria (Fig. 1). Among them, 142 (46.4%) patients received UFH and 164 (53.6%) have received enoxaparin. After PS matching (1:1 ratio), we included 130 patients based on predefined criteria, 65 patients in each group.

### Demographic and clinical characteristics

In the whole cohort, most patients were males (63.5%) with a mean age of 62.9 ± 15.58 years. Diabetes mellitus (65.7%) was the most prevalent co-morbidity, followed by hypertension (60.5%), dyslipidemia (23.9%), and chronic kidney disease (17.3%). Before the PS matching, there were statistically significant differences between the two groups in age and APACHE II score at admission, which were higher in the UFH group (*p*-value =  < 0.01). Moreover, there were significant differences in some laboratory values at baseline, including blood urea nitrogen (BUN), serum creatinine, lactic acid, D-dimer, aPTT, and ferritin levels. Conversely, patients who received enoxaparin had a lower AKI event within the first 24 h of ICU admission than those who received UFH (*p*-value =  < 0.01). Most of these differences were comparable between the two groups after using PS matching, except those who received enoxaparin had a lower baseline of serum creatinine, BUN, and hypertension as comorbid conditions (Table 1).

**Table 1:** Summary of demography and baseline characteristics

	**Before propensity score (PS) matching**				**After propensity score (PS) matching**			
	**Overall (*N* = 306)**	**UFH (*N* = 142)**	**Enoxaparin (*N* = 164)**	***P*-value**	**Overall (*N* = 130)**	**UFH (*N* = 65)**	**Enoxaparin (*N* = 65)**	***P*-value**
**Age (Years), Mean (SD)**	62.9 (15.58)	67.9 (13.66)	58.7 (15.92)	< .01*	59.0 (17.24)	63.7 (12.69)	54.4 (19.85)	0.90^
**Gender – Male, n (%)**	193 ( 63.5)	93 ( 66.4)	100 ( 61.0)	0.32^^	86 ( 66.7)	47 ( 73.4)	39 ( 60.0)	0.10^^
**APACHE II score, Median (Q1,Q3)**	15.0 (10.00, 25.00)	20.0 (15.00, 31.00)	12.0 (8.00, 17.00)	< .01*	15.0 (9.00, 25.00)	17.0 (11.00, 25.00)	12.0 (8.00, 21.00)	0.62^
**Early use of Dexamethasone within 24 h, n (%)**	187 ( 61.1)	87 ( 61.3)	100 ( 61.0)	0.95^^	76 ( 58.5)	37 ( 56.9)	39 ( 60.0)	0.72^^
**Early use of Tocilizumab within 24 h, n (%)**	77 ( 25.2)	29 ( 20.4)	48 ( 29.3)	0.07^^	28 ( 21.5)	14 ( 21.5)	14 ( 21.5)	> 0.99^^
**Proning at admission, n (%)**	68 ( 23.1)	24 ( 17.8)	44 ( 27.7)	0.04^^	29 ( 23.0)	13 ( 21.3)	16 ( 24.6)	0.65^^
**Serum creatinine (mmol/L) at admission, Median (Q1,Q3)**	93.0 (69.00, 161.00)	162.0 (113.50, 276.00)	72.0 (61.00, 88.00)	< .01*	98.0 (69.00, 135.00)	128.5 (97.00, 167.50)	74.0 (63.00, 100.00)	0.02*
**Blood Urea Nitrogen (BUN) (mmol/l) at admission, Median (Q1,Q3)**	8.1 (5.20, 15.10)	14.7 (9.00, 22.30)	5.7 (4.20, 8.10)	< .01*	7.9 (5.10, 13.10)	10.6 (6.70, 15.90)	6.0 (4.30, 8.69)	< .01*
**Acute Kidney Injury (AKI) within 24 h of ICU admission, n (%)**	99 ( 32.8)	82 ( 57.7)	17 ( 10.6)	< .01^^	45 ( 34.6)	30 ( 46.2)	15 ( 23.1)	0.08^^
**Lowest PaO2/FiO2 ratio within 24 h of ICU admission, Median (Q1,Q3)**	85.0 (60.62, 151.40)	85.0 (59.87, 146.20)	85.5 (62.25, 157.70)	0.56^	86.5 (64.77, 165.70)	85.0 (56.00, 144.00)	97.8 (68.90, 202.50)	0.08^
**Highest FIO2 requirement (%) at admission, Median (Q1,Q3)**	70.0 (45.00, 90.00)	72.5 (50.00, 100.00)	70.0 (40.00, 90.00)	0.20^	70.0 (45.00, 95.00)	70.0 (50.00, 100.00)	65.0 (40.00, 90.00)	0.19^
**Vasoactive Inotropic Score, Mean (SD)**	8.4 (46.83)	14.7 (58.97)	3.2 (32.97)	0.05*	6.0 (37.58)	4.5 (12.48)	7.5 (51.64)	0.08^
**Lactic acid baseline (mmol/l), Median (Q1,Q3)**	1.7 (1.32, 2.48)	1.9 (1.31, 2.61)	1.6 (1.33, 2.11)	0.03^	1.6 (1.30, 2.60)	1.9 (1.35, 2.67)	1.6 (1.30, 2.30)	0.16^
**Platelets count baseline (10^9/l), Median (Q1,Q3)**	252.0 (185.00, 323.00)	260.0 (184.00, 320.00)	242.0 (188.00, 326.00)	0.64^	260.5 (190.50, 330.50)	260.0 (184.00, 310.00)	264.0 (199.00, 354.00)	0.50^
**International Normalized Ratio (INR), Median (Q1,Q3)**	1.1 (1.00, 1.20)	1.1 (1.00, 1.23)	1.1 (1.00, 1.19)	0.34^	1.1 (1.00, 1.20)	1.0 (1.00, 1.21)	1.1 (1.02, 1.19)	0.43^
**Activated partial thromboplastin time (aPTT) baseline (Seconds), Median (Q1,Q3)**	29.7 (26.40, 33.60)	29.9 (26.70, 34.50)	29.0 (26.00, 32.20)	0.05^	30.0 (26.70, 34.40)	29.9 (26.40, 33.40)	30.1 (26.70, 34.50)	0.86^
**Total bilirubin (umol/l), Median (Q1,Q3)**	9.4 (6.70, 13.00)	9.3 (6.10, 13.10)	9.4 (6.80, 12.60)	0.58^	9.1 (6.20, 13.00)	9.6 (6.10, 13.00)	8.9 (6.70, 12.80)	0.95^
**Alanine transaminase (ALT) at admission (U/L), Median (Q1,Q3)**	36.0 (24.00, 55.00)	35.5 (24.00, 61.00)	36.0 (25.00, 50.00)	0.85^	36.0 (25.00, 55.00)	36.5 (26.00, 61.00)	34.0 (23.00, 47.00)	0.37^
**Aspartate transaminase (AST) at admission (U/L), Median (Q1,Q3)**	50.0 (33.00, 72.50)	52.0 (34.00, 75.00)	50.0 (32.00, 70.00)	0.31^	50.0 (31.00, 74.00)	53.0 (37.00, 73.00)	41.0 (28.00, 77.00)	0.16^
								^
**Albumin baseline (gm/l), Median (Q1,Q3)**	33.0 (29.00, 36.00)	33.0 (28.00, 36.00)	33.0 (29.00, 36.80)	0.55^	33.0 (29.00, 36.50)	33.0 (29.00, 36.40)	33.0 (29.00, 37.00)	0.95*
**C-reactive protein (CRP) baseline (mg/l), Median (Q1,Q3)**	130.0 (73.00, 197.00)	138.5 (81.50, 202.00)	119.0 (64.43, 182.00)	0.11^	82.0 (37.00, 149.00)	82.0 (62.00, 143.00)	84.0 (22.90, 170.00)	0.61^
**Erythrocyte sedimentation rate (ESR) baseline (mm/hr), Median (Q1,Q3)**	68.0 (41.00, 98.50)	70.0 (43.00, 107.00)	66.0 (35.00, 92.00)	0.32*	66.0 (40.00, 107.00)	79.5 (45.00, 108.00)	54.0 (24.00, 88.50)	0.14*
**Fibrinogen level baseline (gm/l), Median (Q1,Q3)**	5.2 (3.66, 7.37)	5.3 (3.84, 7.22)	5.2 (2.87, 7.56)	0.59*	5.3 (3.27, 7.45)	6.0 (4.03, 7.37)	4.6 (2.60, 7.53)	0.15*
**D-dimer level baseline (mg/l), Median (Q1,Q3)**	1.2 (0.75, 3.40)	2.0 (0.93, 5.12)	1.0 (0.63, 2.08)	0.01^	1.2 (0.68, 3.54)	1.6 (0.82, 4.06)	1.0 (0.57, 2.37)	0.83^
**Ferritin level baseline (ug/l), Median (Q1,Q3)**	797.9 (359.60, 1587.50)	997.7 (456.50, 2047.00)	709.8 (306.20, 1452.00)	0.04^	734.9 (336.60, 1539.00)	823.5 (419.35, 1593.50)	693.7 (267.20, 1472.50)	0.33^
**Blood glucose level baseline (mmol/l), Median (Q1,Q3)**	10.8 (7.80, 15.65)	11.8 (8.00, 16.50)	10.0 (7.40, 14.30)	0.06^	11.0 (8.30, 16.10)	12.8 (8.50, 16.80)	10.7 (8.03, 14.30)	0.31^
**Highest heart rate (HR) at admission (BPM), Median (Q1,Q3)**	103.0 (90.00, 114.00)	104.0 (91.00, 119.00)	101.0 (89.00, 111.00)	0.08^	104.0 (91.00, 115.00)	103.0 (90.00, 118.00)	104.0 (93.50, 114.50)	0.93^
**Maximum body temperature baseline (°C), Median (Q1,Q3)**	37.3 (37.00, 38.00)	37.4 (37.00, 38.00)	37.3 (37.00, 37.90)	0.26^	37.3 (37.00, 38.00)	37.4 (37.00, 38.30)	37.3 (36.90, 37.85)	0.09^
**Aspirin use during ICU stay, n (%)**	84 ( 27.5)	55 ( 38.7)	29 ( 17.7)	< .01^^	32 ( 24.6)	22 ( 33.8)	10 ( 15.4)	0.01^^
**History of Bleeding within 6 months prior ICU admission, n (%)**	1 ( 0.3)	0 ( 0.0)	1 ( 0.6)	0.35**	0 (0)	0 (0)	0 (0)	NA
**Patient received nephrotoxic drugs/material during ICU stay, n (%)**^a^	236 ( 78.4)	122 ( 87.8)	114 ( 70.4)	<0.01^^	100 ( 78.1)	54 ( 85.7)	46 ( 70.8)	0.04^^
**Comorbidity, n (%)**								
Atrial fibrillation (A Fib)	14 ( 4.6)	10 ( 7.0)	4 ( 2.4)	0.05^^	6 ( 4.6)	4 ( 6.2)	2 ( 3.1)	0.40**
Hypertension	185 ( 60.5)	107 ( 75.4)	78 ( 47.6)	< .01^^	75 ( 57.7)	45 ( 69.2)	30 ( 46.2)	0.007^^
Diabetes Mellitus	201 ( 65.7)	106 ( 74.6)	95 ( 57.9)	0.002^^	83 ( 63.8)	43 ( 66.2)	40 ( 61.5)	0.58^^
Dyslipidemia	73 ( 23.9)	37 ( 26.1)	36 ( 22.0)	0.40^^	21 ( 16.2)	11 ( 16.9)	10 ( 15.4)	0.81^^
Chronic kidney disease (CKD)	53 ( 17.3)	51 ( 35.9)	2 ( 1.2)	< .01^^	5 ( 3.8)	3 ( 4.6)	2 ( 3.1)	0.64**
Cancer	12 ( 3.9)	3 ( 2.1)	9 ( 5.5)	0.12^^	3 ( 2.3)	2 ( 3.1)	1 ( 1.5)	0.55**
Deep Vein Thrombosis (DVT)	0 (0)	0 (0)	0 (0)	NA	0 (0)	0 (0)	0 (0)	NA
Pulmonary Embolism (PE)	1 ( 0.3)	1 ( 0.7)	0 ( 0.0)	0.28**	1 ( 0.8)	1 ( 1.5)	0 ( 0.0)	0.31**
Liver disease (any type)	6 ( 2.0)	3 ( 2.1)	3 ( 1.8)	0.85**	3 ( 2.3)	2 ( 3.1)	1 ( 1.5)	0.55**
Stroke	26 ( 8.5)	12 ( 8.5)	14 ( 8.5)	0.97^^	8 ( 6.2)	3 ( 4.6)	5 ( 7.7)	0.46**

^a^Nephrotoxic medications/ material included IV Vancomycin, Gentamicin, Amikacin, Contrast, Colistin, Furosemide, and/or Sulfamethoxazole/trimethoprim

^*^T Test / ^ Wilcoxon rank sum test is used to calculate the *P*-value

^^ Chi square/ ** Fisher’s Exact teat is used to calculate *P*-value

### Thrombosis/bleeding/thrombocytopenia

All thrombosis events were statistically significantly higher among the UFH group compared with patients who received Enoxaparin (18.3% vs. 4.6%; *p*-value = 0.02) in the crude analysis as well in logistic regression analysis (OR: 4.10 (1.05, 15.93); *p*-value = 0.04). Major bleeding and all bleeding cases were similar between the two groups (OR: 1.10 (0.14, 8.56); *p*-value = 0.93 and OR: 0.40 (0.07, 2.29); *p*-value = 0.31, respectively). On the other hand, the blood product transfusion requirement during ICU was higher in the UFH group; however, it failed to reach a statistical significance difference (OR: 2.98 (0.85, 10.39); *p*-value = 0.09). Of note, the use of aspirin during ICU stay was higher in the UFH group than enoxaparin group. The frequencies of HIT and HAT were similar between the two groups (Table 2).

**Table 2:** Clinical outcomes and Complications during ICU stay after PS matching

Outcomes	Number of outcomes/Total number of patients		*P*-value	Odds Ratio (OR) (95%CI)	*P*-value $**
	**Enoxaparin**	**UFH**			
**All thrombosis cases, n(%)**^a^	**3 (4.6)**	**11 (18.3)**	**0.02^^**	**4.10 (1.05,15.93)**	**0.04**
**Major bleeding, n(%)**^a^	**2 (3.1)**	**2 (3.4)**	0.93**	**1.10 (0.14,8.56)**	**0.93**
**All bleeding cases (major and minor), n(%)**^a^	**5 (7.7)**	**2 (3.1)**	0.24**	**0.40 (0.07,2.29)**	**0.31**
**Requiring blood products transfusion during ICU stay, n(%)**^a^	**4 (6.2)**	**10 (16.9)**	**0.06^^**	**2.98 (0.85,10.39)**	**0.09**
**Heparin-associated thrombocytopenia (HAT), n(%)**^a^	**11 (16.9)**	**7 (10.8)**	**0.31^^**	**0.48 (0.16,1.39)**	**0.17**
**Heparin-induced thrombocytopenia (HIT), n(%)**^a^	**1 (1.5)**	**0 (0.0)**	0.32**	**NC**	**NC**
	**Enoxaparin**	**UFH**	**P-value**	**Hazard Ratio (HR) (95%CI)**	***P*-value $**
**30-day mortality, n (%)**^a^	**20 (36.4)**	**27 (57.4)**	**0.03^^**	**0.90 (0.49, 1.64)**	**0.73**
**In-hospital mortality, n (%)**^a^	**21 (38.2)**	**28 (56.0)**	**0.07^^**	**1.18 (0.66, 2.13)**	**0.57**
	**Enoxaparin**	**UFH**	***P*-value**	**beta coefficient (Estimates) (95%CI)**	*P*-value $*
**Ventilator free days, Mean (SD**^a^	**15.3 (13.59)**	**11.8 (12.26)**	**0.08^**	**0.08 (-0.67,0.83)**	**0.83**
**ICU Length of Stay (Days), Median (Q1, Q3)**^a^	**8.0 (5.00, 15.00)**	**11.0 (6.00, 16.00)**	**0.19^**	**0.05 (-0.19,0.29)**	**0.66**
**Hospital Length of Stay (Days), Median (Q1, Q3)**^a^	**13.0 (9.00, 19.00)**	**19.0 (11.00, 25.00)**	**0.01^**	**0.22 (-0.03,0.48)**	**0.09**

*NC* Not computable due to low counts

^a^Denominator of the percentage is the total number of patients

^*^T -Test / ^ Wilcoxon rank sum test is used to calculate the *P*-value

**^^** Chi-square test/** Fisher’s Exact test is used to calculate *P*-value

^$**^ Logistic regression is used to calculate the OR and *p*-value

^$^ Cox proportional hazards regression analysis used to calculate HR and *p*-value

^$*^ Generalized linear model is used to calculate estimates and *p*-value

### Mortality, ventilator free days, and length of stay

Neither the in-hospital mortality (HR: 1.18 (0.66, 2.13); *p*-value = 0.57) nor the 30-day mortality (HR: 0.90 (0.49, 1.64); *p*-value = 0.73) were significantly different between the two groups in Cox hazards regression analysis. Moreover, there was no significant difference in the ICU length of stay and VFDs between the two groups, but the hospital length of stay was longer in patients who received UFH; however, it did not reach the statistically significant (beta coefficient: 0.22; 95% CI: -0.03, 0.48; *p*-value = 0.09) (Table 2).

### Follow-up biomarkers during ICU stay

During the ICU stay, C-reactive protein (CRP) levels as a follow-up biomarker were lower in the enoxaparin group in the crude analysis compared to patients who received UFH. All subsequent biomarkers in the regression analysis, such as D-dimers, Fibrinogen, ESR, and CRP levels were not significant in the regression analysis (Table 3).

**Table 3:** Surrogate markers follow-up (Peak levels) during ICU stay

Surrogate markers	Enoxaparin	UFH	*P*-value ^	beta coefficient (Estimates) (95%CI)	*P*-value $*
**D-dimer level (mg/l), Median (Q1, Q3)**^a^	2.7 (0.89, 18.90)	3.9 (2.01, 17.72)	0.22^	0.16 (-0.42,0.74)	0.59
**Fibrinogen Level, Median (Q1, Q3)**^a^	5.8 (4.33, 8.36)	6.1 (4.59, 7.37)	0.72*	-0.04 (-0.24,0.17)	0.72
**C-reactive protein (CRP), Median (Q1, Q3)**^a^	123.0 (48.30, 231.00)	177.5 (104.00, 258.00)	0.04^	0.17 (-0.16,0.51)	0.31
**Erythrocyte sedimentation rate (ESR), Median (Q1, Q3)**^a^	71.0 (50.00, 108.00)	70.0 (40.00, 102.00)	0.51*	-0.08 (-0.73,0.57)	0.80

^a^Denominator of the percentage is the total number of patients

^*^T -Test / ^ Wilcoxon rank sum test is used to calculate the *P*-value

^$*^ Generalized linear model is used to calculate estimates and *p*-value

## Discussion

This multicenter observational cohort study aimed to investigate which therapy is more effective for critically ill patients with COVID-19 : enoxaparin or UFH. We found that the standard prophylactic enoxaparin dose significantly reduced the risk of arterial or venous thrombosis compared with standard prophylactic UFH dose. Although the number of patients who received UFH required more blood transfusion than the enoxaparin group, there was no difference in minor or major bleeding risk between the two groups. Moreover, no significant difference in the ICU length of stay and VFD between the two groups, but patients on enoxaparin had shorter hospital length of stay compared to the UFH group. In addition, the rate of HIT and HAT were similar in both groups.

In comparison to UFH, enoxaparin has  superior efficacy in the prevention of thromboembolic events with less bleeding risk in critically ill trauma patients, orthopedic surgery patients, and high-risk medically ill patients [27–29]. In our study, the number of venous thromboembolism (VTE) and arterial thromboembolism (ATE) events were statistically significantly lower in the enoxaparin group compared with the UFH group. Although there was no difference in the coagulation profiles between the two groups at baseline, D-dimer and ferritin levels were found to be slightly elevated in the UFH group even after PS matching but did not reach to a statistically significant diffrence. Therefore this was insufficient to draw any conclusions about the relationship between increased thromboembolic events in the UFH group and raised coagulation parameters [30].

The association between high D-dimer levels and adverse outcomes or thromboembolic events has been extensively researched in medical literature [30–32]. Additionally, compared to patients with mild to moderate disease, those with severe COVID-19 illness exhibit hyper-coagulopathy state more frequently [33, 34]. It is interesting to note that numerous studies suggested using therapeutic doses of LMWH or UFH in critically ill COVID-19 patients with increased D-dimer and coagulopathy to prevent thrombosis and improve survival in those populations [35–37]. Additionally,  it is important to note that pre-comorbidities in our cohort such as type 2 diabetes, stroke, cancer, and CKD were not statistically significant in either group.

Our study demonstrated no significant difference between enoxaparin and UFH groups in minor or major bleeding occurrence, and the need for blood products transfusion during ICU stay. The HEP-COVID-19 trial compared the therapeutic-dose LMWH (enoxaparin) versus prophylactic dose heparins (UFH or enoxaparin or dalteparin) among high-risk hospitalized patients with COVID-19 and showed a marked reduction in major VTE, arterial thromboembolism events, and mortality with a similar bleeding rate in the therapeutic-dose LMWH group compared to standard heparin prophylaxis in medically ill COVID-19 patients but not in critically ill patients [9]. The clinical guideline for treating COVID-19 pneumonia recommends using LMWH or UFH at standard prophylaxis doses in hospitalized medical and critically ill patients rather than full therapeutic doses [10].

In our study, there was no difference between the enoxaparin and UFH groups in terms of 30-day mortality or in-hospital mortality. Similar to this, a large systemic review and meta-analysis that examined the effectiveness and safety of intermediate-to-therapeutic versus prophylactic anticoagulation doses in hospitalized COVID-19 patients led to the conclusion that there was no difference in hospital mortality with either intermediate or therapeutic anticoagulation doses compared with standard prophylactic doses [38]. Moreover, our study found no significant difference in ICU length of stay between the two groups, but there was  a difference in hospital length of stay favoring enoxaparin over UFH. Pawlowski et. al. reported shorter hospital and ICU stays among COVID-19 patients who received enoxaparin compared to patients who received UFH. After controlling for confounding variables, the length of ICU stay remained shorter for the enoxaparin group (0.9 vs. 1.4 days), but that was not statically significant [18]. In our study, shorter durations of hospital stay with prophylactic enoxaparin could be attributed to the fact that the enoxaparin group might had less overall complications compared to UFH at baseline.

Patients with COVID-19 who are critically ill experience life-threatening coagulopathies and thromboembolic consequences, which necessitate for aggressive anticoagulation and careful observation. HIT, however, can change the risk–benefit ratio of anticoagulation by raising the risk of serious thrombotic events. According to our data, there was no statistically significant difference between the two groups in the rate  of HIT and HAT. Intensive care units reported a nearly tenfold increased incidence of HIT with severe COVID-19 in one retrospective study that examined all cases of HIT among patients presenting with acute respiratory distress syndrome (ARDS). The timing and exposure to therapeutic doses of UFH, as well as the fact that some patients were receiving extracorporeal membrane oxygenation, were all linked in this study to the HIT occurrence [39]. We used standard doses of UFH in our cohort, which may account for the lack of variation in HIT and HAT incidence between the groups that we found. A recent meta-analysis has confirmed that the rates  of HIT was high in COVID-19 patients receiving therapeutic anticoagulation with UFH [40].

The retrospective study design and the use of administrative data are two limitations of our study. Although we have utilized the PS matching strategy to reduce bias and limit confounding, some unmeasured confounders may still pose a risk. Furthermore, it is questionable whether our results can be generalized to other LMWH agents. Additionally, the choice of the anticoagulant therapy for thromboprophylaxis was left up to the treating clinicians’ judgment and might have been influenced by the constantly evolving national and international COVID-19 management policies. 

## Conclusion

Prophylactic enoxaparin use in critically ill patients with COVID-19 may provided a significant reduction in thrombosis with similar bleeding risk compared to UFH. Moreover, enoxaparin was associated with shorter hospital LOS without mortality benefits. Further randomized clinical and interventional studies are required to confirm our findings.  

## Data Availability

The datasets supporting the conclusions of this article are available from the corresponding author on reasonable request.
